# Assessment of osteoporosis awareness and knowledge levels in people living with HIV

**DOI:** 10.1590/1806-9282.20240925

**Published:** 2025-03-17

**Authors:** Burak Sarikaya, Mustafa Hüseyin Temel, Emre Ata, Vahibe Aydin Sarikaya, Rıza Aytaç Çetinkaya

**Affiliations:** 1 University of Health Sciences, Sultan 2. Abdulhamid Han Training and Research Hospital, Department of Infectious Diseases and Clinical Microbiology – İstanbul, Turkey.; 2 Usküdar State Hospital, Clinic of Physical Medicine and Rehabilitation – İstanbul, Turkey.; 3 University of Health Sciences, Sultan 2. Abdulhamid Han Training and Research Hospital, Department of Physical Medicine and Rehabilitation – İstanbul, Turkey.; 4 Haydarpaşa Numune Training and Research Hospital, Department of Infectious Diseases and Clinical Microbiology – İstanbul, Turkey.; 5 Reyap Health Group, Department of Infectious Diseases and Clinical Microbiology – İstanbul, Turkey.

**Keywords:** Human immunodeficiency virus, Osteoporosis, Awareness, Knowledge

## Abstract

**OBJECTIVE:**

Human immunodeficiency virus is associated with an increased risk of osteopenia and osteoporosis through multiple complex mechanisms. With an increased awareness on this issue, patients can avoid the preventable causes of osteoporosis. The aim of this study was to assess the awareness and knowledge regarding osteoporosis among people living with HIV (PLWH).

**METHODS:**

A total of 329 PLWH agreed to participate in the study. The patients completed a Google Forms questionnaire consisting of three sections. The first section included binary (yes/no) questions to assess participants’ awareness of osteoporosis, knowledge about the condition, perceived susceptibility, and understanding of potential complications. The second section employed the Osteoporosis Awareness Scale, while the third section employed the Revised Osteoporosis Knowledge Tool.

**RESULTS:**

The average age of the 329 patients was 40.21±12.4 years, and 89.4% were male. The mean scores were 66.97±20.68 on the Osteoporosis Awareness Scale and 16.34±6.37 on the Revised Osteoporosis Knowledge Tool. When asked whether they were familiar with osteoporosis, 53.2% of the participants responded yes. Notably, 40% of the participants had knowledge about osteoporosis and its complications, with 50.8% believing that osteoporosis is a serious condition that can be life-threatening. Osteoporosis awareness increased proportionally with the level of education (p<0.001). Patients with a pre-treatment CD4 count<200/mm³ had significantly lower levels of both osteoporosis awareness and knowledge (p<0.001).

**CONCLUSION:**

This study revealed that PLWH have insufficient knowledge and low awareness regarding osteoporosis. In particular, patients with a pre-treatment CD4 count<200/mm³ and those with lower education levels were found to have the lowest awareness level of osteoporosis.

## INTRODUCTION

The widespread use of effective antiretroviral therapy (ART) has allowed people living with HIV (PLWH) to achieve life expectancies similar to those of the human immunodeficiency virus (HIV)-uninfected population^1^.

HIV is associated with an increased risk of osteopenia and osteoporosis^2^. The mechanisms leading to bone diseases in PLWH are complex. In addition to risk factors such as advanced age, low body mass index (BMI), nutritional factors, and toxic habits affecting the general population, PLWH face the risks of developing osteopenia/osteoporosis associated with systemic inflammation due to viral replication and ART use (especially tenofovir disoproxil fumarate with boosters)^3,4^. Moreover, hypogonadism, a significant risk factor for osteoporosis, is highly prevalent in HIV-positive males, reaching up to 20%, which is notably higher compared to the general population^5^.

PLWH have a higher risk of fractures, with a 35-68% increased risk of fragility fractures compared to the general population^3,6^. It is evident that fractures caused by osteoporosis not only harm patients’ health but also lead to socioeconomic losses. To reduce the incidence of osteoporosis, interventions focusing on community-wide measures aimed at mitigating associated risk factors are crucial^7^.

The aim of this study was to quantitatively assess awareness and knowledge levels about osteoporosis among individuals diagnosed with HIV. The study also aimed to evaluate potential differences and correlations in osteoporosis knowledge and awareness levels based on age, gender, educational level, treatments used, viral load, and CD4 count among HIV patients.

## METHODS

This study was conducted with the approval of the Health Sciences University Hamidiye Scientific Research Ethics Committee (14.03.2024-3/36).

The clinical trial was registered as NCT05769413 in a publicly accessible database. The study was conducted at Health Sciences University Sultan II. Abdulhamid Han Training and Research Hospital between March and May 2024. Patients at the HIV outpatient clinic were informed about the research and invited to participate through a Google Forms questionnaire, which required informed consent before completion.

The sample size was determined using the following formula: $n=\frac{Z_{\frac{\alpha }{2}}^{2}xC^{2}}{kD^{2}}\left\{1+\left(k-1\right)\rho \right\}$ , where *k* represents the number of items on the Likert scale, specifically *k*=4 for the Osteoporosis Awareness Scale (OAS). The pairwise correlation coefficient (ρ) was assumed to be 0.5, and the coefficient of variation (*C*) for each Likert-item scale was assumed to be 1. Additionally, *D* was set at 0.10, which represents a 10% relative tolerable error, where $D=\frac{B}{\mu }$ , *B* signifies the bound of error, and μ is the sample mean. The value of $Z_{\frac{\alpha }{2}}$ corresponded to the 100 (1-α/2)th percentile of the standard normal distribution, and for a 95% confidence interval, $Z_{\frac{\alpha }{2}}$ was assumed to be 1.96. Consequently, the required sample size was calculated to be 240. Allowing for a 10% dropout rate, the total recruitment goal was set at 264. Ultimately, 329 patients were included.

The criteria for inclusion were as follows: aged≥18 years, diagnosed with HIV, capable of providing informed consent, fluent in the primary language of the study, and possessed cognitive capacity to complete the study questionnaires and assessments.

The criteria for exclusion were as follows: the presence of known cognitive impairments or dementia, a history of psychiatric disorders impacting study participation or assessment results, and other chronic bone diseases.

To measure the awareness level of osteoporosis, patients were administered OAS, a structured questionnaire designed to assess their understanding of osteoporosis. To investigate participants’ osteoporosis knowledge level, the Revised Osteoporosis Knowledge Tool (OKAT) was employed.

The OAS, developed by Choi et al.^8^, includes 31 items across five sub-dimensions, rated on a 4-point Likert scale. The factors were labeled as "preventive behaviors (10 items)," "risk factors (5 items)," "characteristics of osteoporosis (6 items)," "improving bone health (5 items)," and "bone physiology (5 items)." The cumulative percent of variance was 60.92%, and the eigenvalues ranged from 1.20 to 12.44. The Cronbach's alpha was 0.948 and ranged from 0.804 to 0.917. The potential scores on the OAS range from a minimum of 31 to a maximum of 124. The overall osteoporosis awareness level was reflected in the mean score obtained from both scales, with higher scores indicating greater awareness.

The revised OKAT was introduced by Gendler et al.^9^. This modified test comprises 32 items, each with a possible score range of 0-32. Within this test, there are two distinct sub-dimensions: nutrition and exercise, consisting of 26 and 20 items, respectively, with an overlap of 14 items that are common to both sub-dimensions. A score higher than 25.6 represents a high level of knowledge, a score between 25.6 and 19.2 a moderate level of knowledge, and scores below 19.2 an insufficient level of knowledge.

### Statistical analysis

Statistical analysis was conducted using the IBM SPSS software version 22. The characteristics of quantitative variables were assessed using centralization and variance measurements: mean±standard deviation. Shapiro-Wilk test was used to determine the normality of the distribution. In cases where the assumptions for parametric tests were not met, the Kruskal-Wallis H test (for more than two groups) and the Mann-Whitney U test (for two groups) were employed. Bonferroni's post hoc correction was applied for multiple comparisons between groups. The Spearman's rank correlation test was used to investigate a possible correlation. A significance level of p=0.05 was set for all analyses.

## RESULTS

A comprehensive assessment was conducted on 564 patients potentially eligible for the study. Among these, 235 patients were excluded from the study. Hence, this study ultimately included 329 individuals diagnosed with HIV, consisting of 294 males (89.4%), with the mean age of the patients being 40.2±12.4 years. A history of fractures was reported by 56 participants (17%).

Descriptive statistics such as BMI, bone densitometry results, HIV viral load levels, and CD4/CD8 ratios for the patients are summarized in Table 1.

**Table 1:** Descriptive statistics of patients.

		N	Minimum	Maximum	Mean	SD
Age (year)		329	23	66	40.21	12.46
BMI		329	16.33	36.89	25.14	4.60
L1-L4 T-score		329	-3.0	1.6	-0.7	1.15
Femur neck T-score		329	-1.8	1.4	-1.1	3.69
CD4^+^ value/mm^3^	Total	329	13	1,355	457	302
	<200/mm^3^	91	13	155	95.07	48.98
	>200/mm^3^	238	230	1,355	596.44	237.09
CD8^+^ value/mm^3^		329	350	2,005	982	428.2
HIV RNA value (kopya/mL)		329	5,465	10.000.000	2.661.514	1.440.112
25-OH vitamin D level		191	8.59	27.4	18.16	6.09
Years since the diagnosis of HIV		329	1	8	3.20	2.11
OAS		329	31	122	66.97	20.68
OKAT		329	0	26	16.34	6.37

HIV: human immunodeficiency virus; n: number of patients; SD: standard deviation; L1-L4: Lumbar 1-4 vertebra; BMI: body mass index; OAS: Osteoporosis Awareness Scale; OKAT: Modified Osteoporosis Knowledge Tool.

A statistically significant higher awareness level of osteoporosis was observed in younger patients (p=0.01) (Table 2).

**Table 2:** Awareness of osteoporosis descriptive statistics of all patients.

		Awareness of osteoporosis			
		All patients	Yes	No	p-value
Age, years (mean±SD)		40.21±12.46	38.56±12.57	42.09±12.11	**0.01**
Years since diagnosis (median, IQR [Q1–Q3])		3 (1–5)	3 (1.5–5)	3 (1–5)	0.375
BMI (kg/m²)		25.14±4.6	24.75±4.01	25.57±5.16	0.16
Educational status n (%)	No formal education	28 (8.5)	14 (8)	14 (9.1)	0.17
	Primary school and above	301 (91.5)	161 (92)	140 (90.9)	

SD: standard deviation; IQR: interquartile range; BMI: body mass index. Statistically significant value is denoted in bold.

Patients with pre-treatment CD4 counts<200/mm³ showed significantly lower osteoporosis awareness and knowledge levels based on OAS and OKAT results (p<0.001).

The average score was 66.97±20.68 on the OAS and 16.34±6.37 on the OKAT. Notably, 53.2% of the participants responded yes to the question of "are you familiar with osteoporosis?" 40.0% were knowledgeable about osteoporosis and its complications, and 50.8% believed osteoporosis to be a serious condition that could be life-threatening.

The awareness of osteoporosis increased proportionally with the educational level (p<0.001). Differences were observed in OAS and OKAT scores based on the educational level (p=0.015 and p<0.001, respectively). Higher scores were found in OAS for participants who had completed university education compared to those who had completed high school education (p=0.03), while in OKAT, primary school education had higher scores compared to no formal education (p<0.001), high school education compared to no formal education (p=0.019), and university education compared to no formal education (p=0.008) (Table 3).

**Table 3:** Osteoporosis Awareness Scale and Revised Osteoporosis Knowledge Tool statistics according to the educational level and treatment regimens used by the patients.

	No formal education (n=28)	Primary school education (n=35)	Middle school education (n=21)	High school education (n=84)	University education (n=161)	p-value
OAS (mean)	65.75±15.81	69.00±15.4	59.33±8.94	62.08±21.98	70.30±22.16	**p=0.015**
OKAT (mean)	12.25±4.85	19.23±4.91	14.67±7.73	16.50±7.34	16.57±5.8	**p<0.001**
	TAF/FTC/BIC (n=224)	TDF/FTC+DTG (n=56)	ABC/3TC/DTG (n=14)	TAF/FTC/EVG/c (n=27)	3TC+DTG (n=7)	p-value
OAS (mean)	65.80±19.49	66.45±25.49	68.43±6.19	77.81±24.68	65.29±0.76	0.199
OKAT (mean)	16.02±6.71	16.05±6.01	19.07±3.47	17.33±5.53	20.71±1.89	0.372

OAS: Osteoporosis Awareness Scale, OKAT: Modified Osteoporosis Knowledge Tool, n: number of patients, TAF: tenofovir alafenamide, FTC: emtricitabine, BIC: bictegravir, TDF: tenofovir disoproxil fumarate, DTG: dolutegravir, ABC: abacavir, 3TC: lamivudine, EVG: elvitegravir, c: cobicistat. Statistically significant values are denoted in bold.

Among male patients, those aged 50 years and above had higher osteoporosis knowledge levels compared to those aged below 50 years. There was no difference in osteoporosis awareness levels between these age groups (p<0.001 and p=0.498, respectively).

Patients receiving tenofovir disoproxil fumarate (TDF)-based treatments exhibited the lowest osteoporosis awareness levels.

## DISCUSSION

This study revealed that PLWH have a low level of knowledge (53.2%) and awareness about osteoporosis, thereby demonstrating insufficient knowledge regarding the role of physical activity and calcium intake through diet in preventing the condition. It was observed that the awareness level of osteoporosis was lower among individuals with low educational level, CD4 count<200/mm³, and older age.

Studies indicate that acquiring knowledge about the disease can facilitate early diagnosis and understanding risk factors can enable prevention through adjustments in lifestyle and behavior^10^. The awareness level of osteoporosis among individuals without an HIV diagnosis in studies conducted in Turkey ranged from 60.8 to 97.9%, while globally, it has been reported to be between 81.6 and 96%. These studies also emphasize that the awareness level of osteoporosis is higher among females in the respective populations^11–13^.

In our study, the male patient ratio was found to be 89%, indicating significant heterogeneity. Hsieh et al.^14^ reported similar male patient ratios and awareness levels among HIV patients in their awareness study, whereas the awareness rates were much higher in the study conducted by Karakaş et al.^15^, which focused on conditions like multiple sclerosis with predominantly female patients. We believe that the lower awareness rates in our study compared to the literature may stem from a higher proportion of male patients in the gender distribution and the lower awareness level of osteoporosis among male individuals.

In patients with a pre-treatment CD4 count<200/mm³, both the awareness and knowledge levels of osteoporosis were significantly lower. The literature suggests that low CD4 levels are associated with a late diagnosis, potentially increasing bone loss through heightened HIV-related immunological activity^3^. Considering the low awareness level of their primary disease and delayed presentation to clinicians in this patient group, the lower awareness level of osteoporosis was evaluated as a consistent negative outcome with their overall mental and physical health.

In this study, the average OAS score among HIV patients was determined to be 66.97±20.68. Reviewing studies in the literature on OAS scores, Karagül and Kartaloğlu^16^ reported an average score of 112.45±8.54 among individuals with hypothyroidism and hyperthyroidism, compared to 116.76±9.78 among healthy participants. In a study on patients with spinal cord injury, Şen et al.^17^ found OAS scores of 59.8±11.4 in female patients and 52.5±11.7 in male patients. Uyanık and Erkal^18^ reported an average OAS score of 55.33±17.02 among individuals with chronic diseases. Based on evidence from the literature, it can be concluded that PLWH have lower awareness levels of osteoporosis compared to healthy individuals, a trend observed similarly in patients with other chronic diseases when compared to the general population.

In our study, a positive correlation was observed between osteoporosis awareness and educational levels, consistent with the literature. Satha et al.^19^, in a study involving 144 patients, emphasized that the osteoporosis awareness level increases with higher educational levels. Similarly, Kutsal et al.^20^ indicated that awareness about osteoporosis increases with educational levels. Given these findings, we believe that addressing this gap by focusing on patients with lower educational levels could enhance their awareness and knowledge about osteoporosis.

In a study by Birabaharan et al.^21^, bone densitometry was performed on only 7% of all patients of which 30% of the patients were male aged 50 and above, highlighting low awareness and screening rates for osteoporosis. In our study, it was revealed that among male patients aged 50 and above, there was a higher level of knowledge about osteoporosis, but there was no difference in the awareness level of osteoporosis between age groups. All male patients aged 50 and above underwent bone densitometry according to guideline recommendations, revealing osteopenia based on L1-L4 T-score values. The higher level of osteoporosis knowledge among our patients aged 50 years and above may be associated with clinicians following current guidelines to perform bone screening for all patients in this age group and providing informative education about osteoporosis.

It is known that among the PLWH population, regimens containing boosters, particularly TDF-based medications, are associated with increased osteoporotic risks^21^. Treatment decisions in PLWH involve collaborative decision-making between patients and health-care providers. It has been considered that patients with lower osteoporosis awareness levels might have lower rates of refusal to use TDF, and clinicians may choose TDF-containing regimens more frequently in this patient group compared to others, potentially contributing to this outcome.

There are some limitations in this study to be acknowledged. The study's cross-sectional design limits the ability to establish causality between the variables due to its reliance on a single data collection method, which may not accurately capture the changing nature of osteoporosis awareness levels and related factors. In addition, the study failed to examine potential confounding variables, such as medication compliance, socioeconomic factors, or specific lifestyle habits, which may impact the observed associations between the variables being studied.

## CONCLUSION

According to the present study, PLWH were found to have lower awareness levels of osteoporosis and lack sufficient knowledge about the disease compared to the general population. Specifically, those with pre-treatment CD4 levels<200/mm³ and lower educational levels showed the lowest awareness levels of osteoporosis. The findings of this study emphasize the urgent need for proactive measures, educational campaigns, and tailored interventions to enhance the awareness and knowledge of osteoporosis among HIV-positive patients.
